# Effects of heterotrophic *Euglena gracilis* powder on dough microstructure, rheological properties, texture, and nutritional composition of steamed bread

**DOI:** 10.1016/j.fochx.2024.101754

**Published:** 2024-08-20

**Authors:** Jiangyu Zhu, Yifei Cai, Yan Xu, Xiao Wei, Zhengfei Yang, Yongqi Yin, Minato Wakisaka, Weiming Fang

**Affiliations:** a School of Food Science and Engineering, Yangzhou University, No. 196 Huayang West Road, Hanjiang District, Yangzhou 225127, China; b Food Study Centre, Fukuoka Women's University, 1-1-1 Kasumigaoka, Fukuoka 813-8529, Japan

**Keywords:** *Euglena gracilis*, Dough, Chinese steamed bread, Rheological behavior, Nutritional quality

## Abstract

This study investigated the effects of incorporating different levels of *Euglena gracilis* microalgae powder (MP) on the dough properties, rheology, and quality attributes of Chinese steamed bread (CSB) for the first time. Moderate levels of MP (2%) reinforced the gluten network and improved protein structure, while higher levels (4–8%) adversely affected the gluten network and rheological properties. The addition of MP decreased the specific volume, pore number, and pore density of CSB, but increased pore size, hardness, and chewiness. It also imparted a yellow color to the CSB and slowed down moisture loss during storage. Notably, MP effectively increased the protein and lipid content of CSB, enhancing its nutritional value. The results suggest that optimizing the MP level is crucial to achieve nutritional enhancement while maintaining desirable texture and sensory attributes. An addition of 2% MP can strike a balance between nutrition and the overall quality of the final product.

## Highlights

• Incorporation of microalgae powder (MP) affected dough properties and rheology.
• Moderate levels (2%) of MP improved improved gluten network and protein structure.
• MP fortification enhanced nutritional value by increasing protein and lipid content.
• Higher MP levels disrupted gluten network, altering dough rheology and texture.
• Optimum 2% addition balanced nutrition and desirable texture of steamed bread.

## 1 Introduction

In recent years, there has been a growing interest in incorporating microalgae into food products due to their potential as sustainable sources of high-quality proteins, lipids, vitamins, and other bioactive compounds (Caporgno & Mathys, 2018). Microalgae have garnered significant attention as functional ingredients for fortifying various food matrices, owing to their unique nutritional profiles and potential health-promoting properties (Zhu, Xiao, et al., 2024). Among the diverse microalgal species, *Euglena gracilis*, a freshwater microalga, has emerged as a promising candidate for food applications. *E. gracilis* is known for its ability to accumulate valuable biomolecules, such as paramylon (a β-1,3-glucan), proteins, lipids, and antioxidants.

Bread and other baked goods constitute a major component of the human diet worldwide, providing essential nutrients and energy. However, these products are often deficient in certain essential nutrients, particularly proteins and healthy lipids (Guo et al., 2022). Fortifying flour products with microalgal biomass presents an opportunity to enhance their nutritional value while potentially improving their functional and sensory properties. Chinese steamed bread (CSB), a traditional fermented and steamed product, is a staple food in China and other Asian countries. CSB is widely consumed due to its soft and fluffy texture, as well as its convenience and affordability (Wang et al., 2017). Incorporating microalgal biomass into CSB formulations can introduce several challenges due to the potential interactions between the microalgal components and the complex dough system. The gluten network, formed by the interactions between gliadin and glutenin proteins in wheat flour, plays a crucial role in determining the rheological properties, gas-holding capacity, and final product quality of CSB (Liu et al., 2021). The addition of microalgal biomass may influence the gluten network formation, dough rheology, and ultimately, the texture and sensory attributes of the final product.

Despite the potential nutritional benefits and challenges associated with incorporating microalgal biomass into baked goods, none research has been conducted on the impact of *E. gracilis* powder on the physicochemical properties, dough rheology, and quality characteristics of CSB. The current research mainly focuses on the nutritional enhancement of common edible algae such as *Spirulina* and *Chlorella* added to bread and cookies (Caporgno & Mathys, 2018), while lacking an investigation into the effects of other edible algae, such as *E. gracilis*, on the properties of dough and the texture of the final products.

Therefore, this study aims to bridge this knowledge gap by investigating the effects of varying levels of *E. gracilis* microalgae powder (MP) on the dough properties, rheological behavior, and final product quality of CSB. Specifically, the objectives of this study are to: evaluate the influence of MP addition on the pH, total titratable acidity, microstructure, and secondary protein structure of the dough; characterize the changes in dough rheological properties induced by MP incorporation; assess the impact of MP on the color, texture, specific volume, pore distribution, and water activity of the final CSB product; determine the changes in the nutrient composition (protein, lipids, carbohydrates) of CSB resulting from MP fortification; identify the optimal level of MP addition to achieve nutritional enhancement while maintaining desirable physicochemical and sensory properties of CSB.

This study will provide valuable insights into the potential of utilizing *E. gracilis* biomass as a functional ingredient in traditional baked goods, contributing to the development of nutritionally enhanced and sustainable food products.

## 2 Materials and methods

### 2.1 Microalga and materials

The freshwater microalga *Euglena gracilis* (FACHB-849) was purchased from the Freshwater Algae Culture Collection at the Institute of Hydrobiology, National Aquatic Biological Resource Center. The *E. gracilis* was inoculated in HUT medium for heterotrophic cultivation. Cell biomass was evaluated through cell counting under a biological microscope (LM31, Mshot, Guangdong, China) and dry weight method. When the culture reached the late logarithmic phase, it was harvested by centrifugation. The freshly harvested *E. gracilis* was rapidly frozen in liquid nitrogen, followed by freeze-drying for 24 h using a TF-HFD-6 freeze dryer (Tianfeng Industrial Company, China). The freeze-dried microalgae powder (MP) was then sealed and stored at 4 °C for further use. Active dry yeast (*Saccharomyces cerevisiae*) was obtained from Angel Yeast Co., Ltd. (Yangzhou, China). Multipurpose wheat flour were provided by Yihai Jiali Grain and Oil Co., Ltd. (Nanjing, China).

### 2.2 Dough and CSB preparation

The *E. gracilis* powder was weighed in proportions of 0.0%, 2.0%, 4.0%, 6.0%, and 8.0%. Yeast and sugar were weighed in proportions of 1.5% and 2.5% respectively. Wheat flour was also weighed to make a total weight of 300 g. 150 g of water was weighed and yeast and sugar were added to activate at a temperature of 37 ± 2 °C. The mixed flour and yeast mixture were then poured into a dough mixer (SM-1210CL, Netmego, China) and stirred at a slow speed of 60 rpm for 2 min to incorporate the ingredients and then at a high speed of 120 rpm for 13 min to develop the gluten network. This ensured the dough was adequately mixed and developed the necessary gluten network. After shaping, the dough was placed in a proofing cabinet (SRP—18S, bakestar, China) at a temperature of 35 °C and relative humidity of 80% for 60 min. The dough was then rolled and divided into 50 g portions, which were shaped into *E. gracilis* dough and placed in a tray. The *E. gracilis* CSB dough was placed in the proofing cabinet again and proofed at a temperature of 36 °C and humidity of 70% for 20 min to form *E. gracilis* dough. The *E. gracilis* dough was steamed for 20 min on a 2200 W electromagnetic stove (C22-IH110ET, SUPOR, China) to obtain CSB with *E. gracilis*.

### 2.3 pH and Total titratable acidity (TTA)

The method of Buddrick et al. (2014) was used for pH and TTA determination with some modifications. 10 g of fully proofed dough was taken and homogenized in 90 mL of distilled water (cooled to room temperature) using an adjustable high-speed homogenizer (FSH-2 A, Anqing Jiejia Instrument Equipment Co., Ltd., China). The homogenized solution was then centrifuged at a speed of 6000 rpm and the pH of the supernatant was measured using a pH meter (PHS-2F, Shanghai Yeidian Technology Co., Ltd., China). The supernatant was titrated with 0.01 mol/L NaOH solution to a pH of 8.6 ± 0.1, and the volume of NaOH consumed (mL) was recorded as the TTA of the dough.

### 2.4 Microstructural determination of the dough

Microstructure of the dough was determined by the scanning electron microscope (SEM). Frozen dried dough was cut into small pieces with a flat surface of about 5 mm^3^, and the samples were fixed on the sample stage using conductive adhesive tape. After gold coating treatment with a Hitachi IB-5 ion coater (SCD 500, Hitachi Ltd., Japan), the samples were observed and photographed under a field emission scanning electron microscope (S-4800II, Hitachi Ltd., Japan) at an accelerating voltage of 25 kV, with a magnification of 3000 times.

### 2.5 Determination of secondary structure of protein in dough

Secondary structure of protein in dough was determined according to Liu et al. (2017) with some modifications. The freeze-dried dough powder and KBr were mixed in a ratio of 1:100 in the research bowl, fully mixed and ground, and pressed into semi-transparent sheets using an infrared special press. The sheets were scanned in the 400–4000 cm^−1^ range with a micro-FTIR spectrometer (670-IR + 610-IR, Varian Corporation, A) with a resolution of 4 cm-1 for 64 scans. Each sample was repeated three times. The amide I band absorption peak (1600 cm^−1^-1700 cm^−1^) was captured using OMNIC 8.0 software, and the data were baseline-corrected and curve-fitted using Peak Fit software with Gaussian deconvolution technique and second derivative method, with R^2^ > 0.99. The distribution chart of protein secondary structure was obtained, and the relative content of α-helix, β-turn, β-sheet, and irregular curl in the sample was calculated based on the integrated area.

### 2.6 Rheological characterization of the dough

Five grams of fully proofed dough was placed in a parallel plate rheometer (MCR 72, Anton Paar, Austria) for measurement. The plate had a sandblasted surface to ensure no slippage between the dough sample and the rheometer plate during measurements. The diameter of the plates was 40 mm, and the gap between the two plates was set to 3 mm. Excess sample was trimmed from the edges, and dimethyl silicone oil was applied around the exposed sample edge to prevent moisture evaporation and reduce experimental errors. The measurement procedure and conditions were adapted from Correa et al. (2010) and Cao et al. (2019) with some modifications. After lowering the upper plate, the sample was allowed to equilibrate for 10 min at 25 °C to eliminate residual mechanical stress in the dough that could affect the results (Jekle & Becker, 2012). Frequency sweep tests were conducted with a strain of 0.1%, temperature of 25 °C, and frequency ranging from 0.1 to 10 Hz (Cao et al., 2019). The storage modulus (G') and loss modulus (G") of the sample as a function of frequency were obtained. The loss tangent (tan δ) was calculated by dividing G" by G'. The power law parameters were determined by fitting the frequency sweep data to the power law model (G' = K'ω^n’^ and G" = K″ω^n”^). Where ω is the angular frequency, K′ and K″ are consistency indices, and n’ and n” are the power law exponents for G' and G" respectively. The power law exponents (n’ and n”) and the consistency indices (K′ and K″) of G' and G", were calculated for each sample to quantify the effect of algae addition on dough rheology.

### 2.7 Color measurement of CSB

A colorimeter (NH310, Shenzhen Sannes Technology Co., Ltd., China) was used to assess the CSB (Zhu, Fang, et al., 2024). Prior to measurement, the instrument was calibrated, and the internal cut and surface of the CSB were measured under similar light conditions at room temperature. Each sample group was measured three times. The measured parameters included L*, a*, and b* values, where L* represents lightness (brightness) in black and white, with higher values indicating greater whiteness/brightness. The a* value represents the red-green value, decreasing from red to green, and the b* value represents the yellow-blue value, decreasing from yellow to blue. The total color difference of the sample was calculated according to Eq. (1):

(1) $$\mathrm{\Delta E}*={\left[{\left(\mathrm{\Delta L}*\right)}^{2}+{\left(\mathrm{\Delta a}*\right)}^{2}+{\left(\mathrm{\Delta b}*\right)}^{2}\right]}^{\frac{1}{2}}$$

### 2.8 Water activity determination of CSB

Prepared CSB was cooled at room temperature, and the core was crumbled for water activity (Aw) measurement using a water activity meter (HD-3 A, Wuxi HuaKe Instrument Co., Ltd., China) after calibration with sodium chloride and magnesium chloride. Measurements were taken after 1 h of steaming and storage at 4 °C for 24, 72, 120, and 168 h.

### 2.9 Pore distribution on the cross-section of CSB

The prepared CSB was cooled at room temperature and cut into uniform thin slices of 20 mm thickness. The cross-section of the CSB was photographed using a digital camera (EOS 80D, Canon, Japan) to obtain pore images. Image analysis using Image J software (open source) was performed to calculate the average pore number (APN), porosity (PO), average pore area (APA), and pore density (PD) using the following Eqs. (Barsanti et al., 2022; Bartkiene et al., 2013; Buddrick et al., 2014):

(2) $$\begin{array}{c} \mathrm{PO}\;\left(\%\right)=\frac{\text{Pore area}\;\left({\mathrm{cm}}^{2}\right)}{\text{Total area}\;\left({\mathrm{cm}}^{2}\right)}\times 100 \end{array}$$

(3) $$\begin{array}{c} \mathrm{APA}\;\left(10^{-3}\cdot {\mathrm{cm}}^{2}\right)=\frac{\text{Pore area}\;\left({\mathrm{cm}}^{2}\right)}{\text{Number of pores}\;\left(\mathrm{pcs}\right)}\times 1000 \end{array}$$

(4) $$\begin{array}{c} \mathrm{PD}\;\left(\mathrm{pcs}\cdot {\mathrm{cm}}^{-2}\right)=\frac{\text{Number of pores}\;\left(\mathrm{pcs}\right)}{\text{Total area}\;\left({\mathrm{cm}}^{2}\right)} \end{array}$$

### 2.10 Texture profile analysis of CSB and specific volume determination

Following the method of Guo et al. (2022), the prepared CSB was naturally cooled at room temperature and cut into uniform slices with a thickness of 20 mm and a diameter of 40 mm for texture analysis using a texture analyzer (TA-3000, Jinan Saicheng Electronic Technology Co., Ltd.). The parameters of the texture analyzer were set as follows: P36R probe, compression ratio of 50%, testing force of 500 N, testing speed of 3 mm/s, and an interval time of 3 s with a trigger force of 5 g.

The volume of the CSB was measured by displacing rapeseed in a beaker, and the weight of the CSB was measured. Three parallel samples were taken for each group, and the specific volume (SV) of the CSB was calculated using Eq. (5):

(5) $$\begin{array}{c} \text{Bulk density}\;\left(\mathrm{mL}/g\right)=\frac{\text{Volume}\;\left(\mathrm{mL}\right)}{\text{Mass}\;\left(g\right)} \end{array}$$

### 2.11 Nutrient composition analysis of CSB

The protein, fat and moisture contents of the CSB were determined following the official AOAC methodology (AOAC, 2016). The protein content was analyzed using a Kjeldahl nitrogen analyzer, lipids were extracted and determined using ether in a Soxhlet apparatus, and the moisture content was determined in a drying oven. The determination of total available carbohydrate content was conducted according to Carocho et al. (2020). The samples were added to perchloric acid and left to digest overnight. After filtration, anthrone was added to the test tube, boiled and cooled, and detection was performed at 630 nm with a spectrophotometer. The content of each nutrient was expressed as g/100 g of fresh weight. The total calorie of the CSB (Kcal/100 g) was calculated based on the energy conversion coefficient of the nutrient composition.

### 2.12 Statistical analysis

Three repetitions were set for each experiment, and the data obtained were processed using Microsoft Office Excel 2021. Origin 2018 and SPSS 19.0 software were used for data plotting and statistical analysis, and the results were expressed as the mean ± standard deviation (T ± SD). One-way analysis of variance (ANOVA) was used to determine whether there were significant differences in the data for each group. Tukey's (homogeneity of variance) and Dunnett's T3 (unequal variance assumed) multiple comparisons were used to test for significance at the 0.05 level (*p* < 0.05). The relevance among various indicators was determined using the Pearson correlation coefficient method (Nash et al., 2006).

## 3 Results and discussion

### 3.1 Effects of MP on dough pH and TTA

The different amounts of MP added to the dough have an impact on the pH and TTA values as shown in Table S1. The pH of MP dough remains stable at around 5.3, with little variation upon addition of *E. gracilis* powder. This indicates that the addition of MP does not significantly affect the dissociation of H+ during dough fermentation, as various compounds present in wheat flour, such as phosphates, proteins, and amino acids, act as buffers to neutralize acids and bases, maintaining a relatively stable pH environment. However, the TTA value of the dough gradually increases with the addition of *E. gracilis* powder. This is primarily due to the rich organic acids naturally present in *E. gracilis* (Tomita et al., 2016). Furthermore, the abundant nutrients in MP provide additional raw materials for further microbial fermentation, metabolizing these nutrients into organic acids such as acetic acid and lactic acid, resulting in an overall increase in TTA (Li et al., 2014). The inclusion of MP can enhance the number and variety of organic acids in the dough, potentially further affecting the rheology, gas production, and final product quality (Bartkiene et al., 2013).

### 3.2 Effect of MP on dough microstructure

**Fig. 1:**
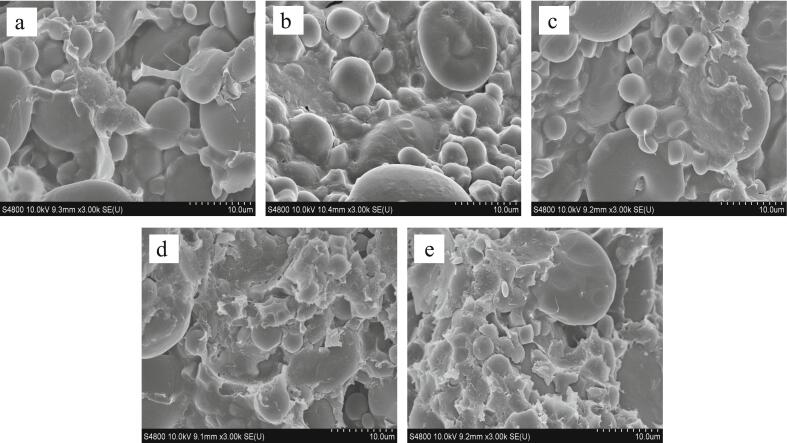
Microstructure of dough with different amount of MP inclusion. (a-e: 0%, 2%, 4%, 6% and 8% MP additions, respectively).

The influence of different dosages of MP on the microstructure of dough was observed using SEM, and the results are shown in Fig. 1. The dough is a system composed of gluten protein network and starch granules embedded in the gluten protein (Han et al., 2020). The gluten protein network is formed by the interaction forces between glutenin and gliadin, such as disulfide bonds, hydrogen bonds, and ionic bonds (Wieser, 2007). Under the SEM, starch granules are spherical or elliptical, while *E. gracilis* cells are spherical and spindle-shaped, both embedded in the gluten protein network. The dough without MP exhibits obvious starch granules and a hard, dense gluten network. As the amount of MP added increases, changes occur in the gluten protein network. In the dough without MP (Fig. 1a), the gluten protein network appears to be in a cohesive cross-linked state, but with some fractures. In the dough with 2% MP added (Fig. 1b), most of the starch granules and algae powder granules are encapsulated in the gluten network, indicating that the appropriate amount of MP added improves the gluten network structure of the dough. When more MP is added (Fig. 1c-e), the molecular disulfide bonds between gliadin and glutenin are disrupted, and the gluten structure cannot completely encapsulate the granules of starch and MP, leading to their exposure. The gluten protein network gradually becomes rough and no longer intact, and the gluten structure in the dough also severely fractures with the increasing amount of MP added.

### 3.3 Effect of MP on secondary structure of dough protein

The secondary structure of proteins in dough plays a crucial role in determining the strength and rheological properties of the gluten network. The addition of microalgae powder can alter the secondary structure of proteins, affecting the overall dough behavior, and the results are summarized in Table 1. The α-helix content remained relatively stable across all dough samples, indicating that the addition of microalgae powder did not significantly affect the ordered α-helical structure of gluten proteins. The β-sheet content initially increased with the addition of 2% microalgae powder, suggesting an enhancement in the ordered β-sheet structure. However, further increasing the microalgae powder led to a gradual decrease in β-sheet content, potentially due to the disruption of intermolecular interactions and the weakening of the gluten network (Cao et al., 2019). The random coil content, representing the disordered structure, did not exhibit significant differences among all treatment groups. Interestingly, the β-turn content showed a decreasing trend with the addition of 2% MP, although the difference was not statistically significant. However, further increasing the MP levels led to a significant increase in β-turn content. β-turns are considered intermediate structures between ordered and disordered conformations, indicating that higher levels of MP might disrupt the native gluten protein structure, leading to a partial unfolding or rearrangement of the polypeptide chains (Khatkar et al., 1995).

**Table 1:** Effects of different inclusion levels of MP on protein secondary structure in dough.

MP addition	α-helix (%)	β-sheet (%)	Random coil (%)	β-turn (%)
0%	18.83 ± 0.21^a^	27.82 ± 0.41^ab^	17.73 ± 0.39^a^	35.63 ± 0.66^ab^
2%	18.53 ± 0.15^a^	28.80 ± 0.44^a^	17.48 ± 0.14^a^	35.19 ± 0.5^b^
4%	18.48 ± 0.15^a^	27.38 ± 0.41^b^	17.32 ± 0.16^a^	36.82 ± 0.68^ab^
6%	18.55 ± 0.02^a^	27.45 ± 0.84^ab^	17.36 ± 0.11^a^	36.63 ± 0.93^ab^
8%	18.81 ± 0.08^a^	26.63 ± 0.30^b^	17.35 ± 0.10^a^	37.21 ± 0.47^a^

***Note*:** Values are expressed as mean ± standard deviation. Different letters within the same column indicate significant differences (p < 0.05).

The observed changes in the secondary structure of gluten proteins can be attributed to the interactions between microalgae components and wheat gluten proteins, as well as the potential dilution or steric effects caused by the incorporation of microalgae powder. The initial increase in β-sheet content at moderate microalgae powder levels suggests a stabilizing effect on the gluten network through intermolecular interactions. However, excessive levels of microalgae powder might disrupt the gluten network, leading to partial unfolding or conformational changes in gluten proteins and a consequent weakening of the overall structure. It is important to note that the secondary structure of proteins is closely related to their functional properties, such as water absorption, viscoelasticity, and gas-holding capacity (Kontogiorgos, 2011; Wieser, 2007). Therefore, the observed changes in the secondary structure of gluten proteins due to microalgae powder addition can have significant implications for the rheological behavior and final product quality of the dough.

### 3.4 Effect of MP on the rheological properties of the dough

**Fig. 2:**
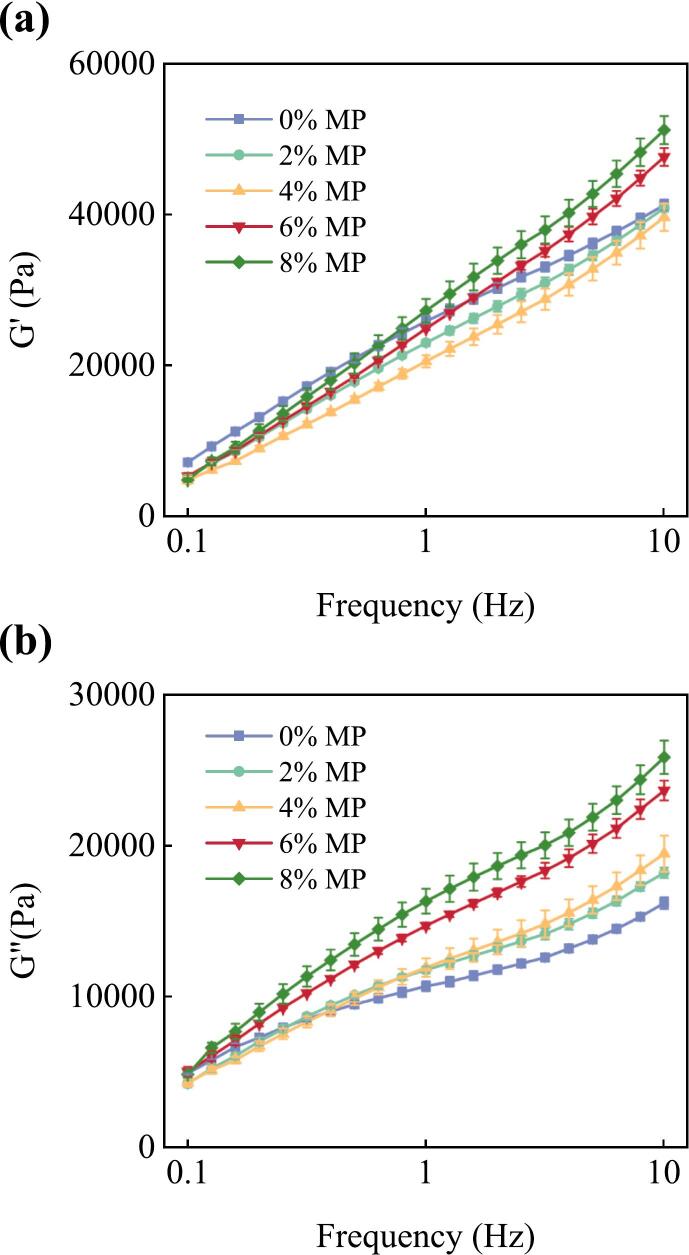
Effects of MP addition on the dough's dynamic rheological properties. (a): the storage modulus G', (b): the loss modulus G".

The rheological properties of dough provide valuable insights into its behavior during processing and are closely related to the final product quality. The dynamic oscillatory measurements, including the storage modulus (G'), loss modulus (G"), and loss tangent (tan δ), offer crucial information about the viscoelastic properties of the dough system, and the results are illustrated in Fig. 2. The storage modulus (G') was consistently higher than the loss modulus (G") across all dough samples, indicating a predominant elastic behavior over viscous behavior. This is a characteristic of solid-like materials and is attributed to the gluten network formed by intermolecular interactions between gluten proteins (Falcão-Rodrigues et al., 2005). Both G' and G" showed an upward trend with increasing frequency, which is typical behavior for viscoelastic materials. The addition of microalgae powder led to a gradual increase in both G' and G" values compared to the control dough, suggesting a reinforcement effect on the overall viscoelastic properties of the dough. The loss tangent (tan δ = G”/G'), which represents the ratio of viscous to elastic behavior, exhibited an increasing trend with increasing microalgae powder addition. A lower tan δ value indicates a more elastic and solid-like behavior, while a higher value suggests a more viscous and liquid-like behavior (Wang et al., 2023). The control dough had the lowest tan δ values across the entire frequency range, indicating a stronger elastic behavior compared to doughs containing microalgae powder. As the microalgae powder addition increased, the tan δ values gradually increased, suggesting a shift towards a more viscous and less elastic behavior.

The power law parameters derived from the frequency sweep tests are presented in Table S2. These parameters provide additional insights into the rheological behavior of the dough samples with varying MP content. As the MP content increased from 0% to 8%, we observed significant changes in these parameters. Both n’ and n” consistently increased with rising MP concentration, indicating that the dough became more frequency-dependent as more algae was added. Notably, n’ remained consistently higher than n” for all concentrations, which is typical for viscoelastic materials. The most substantial increase in both n’ and n” occurred between 0% and 2% MP, suggesting that even a small addition of algae has a significant impact on the dough's rheological behavior. The rate of increase in n’ and n” slowed down at higher algae concentrations, indicating a possible saturation effect. This trend suggests that the addition of MP makes the dough more sensitive to changes in frequency, potentially indicating a less stable network structure at higher algae concentrations. The consistency coefficient K′ and K″ showed an U-shaped trend, initially decreased, followed by a rebound. Notably, even at 8% MP concentration, K′ did not fully recover to its original level seen in the control sample, whereas the K″ value at 8% MP surpassed the initial level of the control sample. This indicates that the viscous component of the dough not only recovered but surpassed its original strength at high MP concentrations.

The observed changes in rheological properties can be attributed to the interactions between microalgae components and gluten proteins, which can disrupt the gluten network structure. The addition of microalgae powder might interfere with the formation and stabilization of intermolecular interactions (e.g., disulfide bonds, hydrogen bonds) within the gluten network, leading to a weaker and less elastic dough structure (Liu et al., 2021). Furthermore, the presence of microalgae components, such as proteins, polysaccharides, and other biomolecules, could act as diluents or fillers, disrupting the continuous gluten network and hindering the development of a strong and cohesive structure (Huang et al., 2019).

It is important to note that the rheological properties of dough are closely related to its handling, machinability, and the final product quality. A well-developed and elastic gluten network is essential for adequate gas retention, dough stability, and the desired texture and volume of the final product (Ortolan & Steel, 2017). Therefore, optimizing the level of microalgae powder addition is crucial to maintain desirable rheological properties while benefiting from the nutritional and functional advantages of microalgae incorporation.

### 3.5 Effect of MP on the color of CSB

Color is an important factor in evaluating the quality of CSB. The addition of MP significantly influenced the color of the CSB core and crust (Table S3). As the MP level increased from 2% to 8%, the L* value (lightness) decreased, while the a* (redness) and b* (yellowness) values increased, resulting in a more pronounced yellow color in the CSB. This color change can be attributed to the presence of carotenoid pigments, such as lutein and β-carotene, in the *E. gracilis* cells (Zhu et al., 2021). These pigments are responsible for the characteristic yellow-to-orange hue observed in the microalgae powder. The crust of the CSB had a lower L* value and higher a* and b* values compared to the core, which may be attributed to the higher temperature of the crust during steaming, leading to increased Maillard browning and non-enzymatic browning reactions (Çelik & Gökmen, 2020; Helou et al., 2016). The total color difference (ΔE) is used to represent the color difference, with ΔE > 3 indicating a significant color difference perceptible to the human eye (Goswami et al., 2015). As shown in Table S3, compared to the control group, the ΔE values for both the crumb and crust of CSB containing MP were >3, indicating a significant color difference from the control group. The higher the MP addition level, the greater the color difference.

It is noteworthy that the heterotrophic nature of *E. gracilis* played a crucial role in obtaining an acceptable color for the CSB. Unlike autotrophic microalgae species that contain significant amounts of chlorophyll, resulting in an undesirable green color, heterotrophic microalgae can suppress chlorophyll production (Zhu, Xiao, et al., 2024). This characteristic allowed the incorporation of microalgae biomass without imparting an off-putting green hue to the CSB, thereby enhancing consumer acceptability.

### 3.6 Water activity changes of CSB during storage

Aw is a critical parameter that significantly influences the physicochemical and sensory properties of baked products, including texture, staling rate, and microbial stability. The Aw values of CSB samples were determined at the time of preparation and after 1, 3, 5, and 7 days of storage to monitor the changes in water mobility and availability.

As shown in Fig. S2, the initial Aw of freshly prepared CSB ranged from 0.972 to 0.984, with the control sample (0% MP) exhibiting the lowest value of 0.972. During the storage period, the Aw of all CSB samples decreased to varying degrees, indicating a gradual loss of moisture and a reduction in water mobility. This decrease in Aw is primarily attributed to the migration and redistribution of moisture within the product, as well as the potential occurrence of starch retrogradation and gluten network rearrangement, which can alter the water-binding capacity of the system (Purhagen et al., 2012). Notably, the rate of Aw decrease was relatively slower for CSB samples containing microalgae powder compared to the control. After 7 days of storage, the Aw of the control sample (0% MP) decreased by 4.2%, while the Aw reduction for samples with 2%, 4%, 6%, and 8% MP was 0.9%, 2%, 2.2%, and 2.8%, respectively. This slower rate of Aw decrease in 2% MP-enhanced CSB can be attributed to the presence of hydrophilic compounds, such as polysaccharides and proteins, which can enhance the water-holding capacity of the system (Sousa et al., 2008). Additionally, the incorporation of microalgae powder might lead to structural changes in the gluten network and starch matrix, potentially altering the water distribution and mobility within the system (Dufour et al., 2024).

Maintaining a higher Aw during storage can have implications for the textural properties and shelf-life of CSB. Higher Aw levels are typically associated with a softer, fluffier texture, but also increase sensitivity to microbial spoilage (Rahman et al., 2022). Therefore, the incorporation of microalgae powder might contribute to maintaining the freshness and optimal taste of CSB during storage, although it is crucial to strike a balance between Aw and microbial stability.

### 3.7 Effects of MP on pore distribution in CSB

The pore distribution in CSB is the result of the collective interaction of various components, with a desirable CSB exhibiting small, densely packed, and uniformly distributed pores. The incorporation of MP led to a decrease in the APN and PD, while increasing the PO and APA in the CSB, resulting in a more coarse and sparsely distributed pore structure. As shown in Table S4, the APN in CSB significantly decreased from 265.00 (control) to 185.67 with the addition of 8% MP. Similarly, the PD, representing the number of pores per unit area, exhibited a decreasing trend, from 29.44 pcs·cm^−2^ in the control to 20.63 pcs·cm^−2^ with 8% MP inclusion. This reduction in pore number and density can be attributed to the dilution effect of MP on the wheat flour proportion, leading to a decreased gluten matrix in the CSB dough. The diminished gluten network weakens the dough's gas-holding capacity and mechanical strength during proofing and steaming, resulting in fewer and fewer pores being formed (Dufour et al., 2024). Conversely, the APA increased from 7.70 × 10^−3^·cm^2^ in the control to 12.47 × 10^−3^·cm^2^ with the addition of 8% MP. This trend indicates that the pores became larger and more coarse as the MP level increased. The PO, representing the percentage of air space in the CSB, exhibited an initial increase from 22.67% (control) to 26.31% with 2% MP. This suggests that moderate levels of MP might promote gas production and retention during proofing, leading to a higher porosity. Excessive MP content, however, can interfere with the formation and stabilization of the gluten network (Dufour et al., 2024; Zhu, Xiao, et al., 2024), compromising the dough's gas-holding capacity, resulting in a decrease in porosity, yet still significantly higher than the control group level.

It is noteworthy that the pore distribution in baked products like CSB plays a crucial role in determining the texture, mouthfeel, and overall sensory acceptance. A uniform distribution of small, densely packed pores is typically associated with a soft, tender texture, while larger and more sparsely distributed pores can lead to a coarser, drier, and less desirable texture (Guo et al., 2022). Therefore, optimizing the MP addition level is essential to strike a balance between maintaining an acceptable pore structure and leveraging the nutritional and functional benefits of microalgae incorporation.

### 3.8 Effects of MP on texture and SV of CSB

**Fig. 3:**
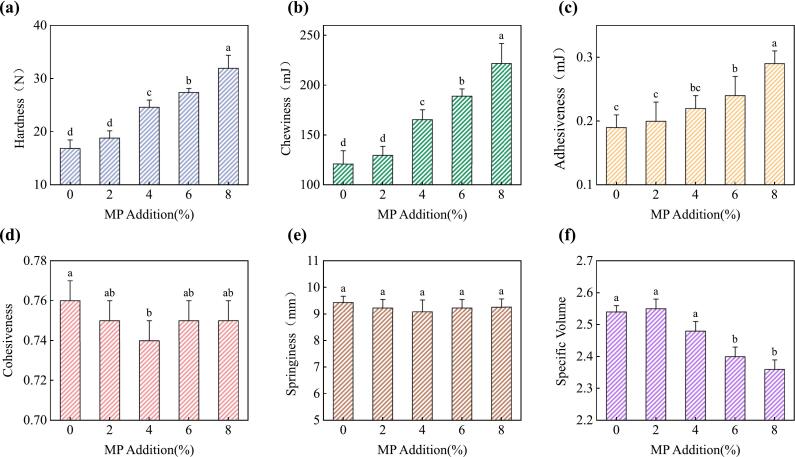
Effects of MP addition on texture profile and SV of CSB. (a): Hardness, (b): Chewiness; (c) Adhesiveness; (d) Cohesiveness; (e): Springiness; (f): SV. Different letters indicate significant differences (*p* < 0.05).

The texture profile analysis (TPA) provides valuable information about the mechanical properties and sensory characteristics of CSB. The incorporation of MP had a significant impact on the textural attributes of the CSB, as shown in Fig. 3. Hardness and chewiness exhibited a significant increasing trend with higher MP levels. Compared to the control, the hardness and chewiness of CSB increased by 1.89 and 1.83 times with the addition of 8% MP, respectively. Higher hardness values indicate a firmer and more resistant texture, while increased chewiness correlates with a denser and more compact crumb structure (Gómez et al., 2008). This can be attributed to the dilution effect of MP on the gluten network, leading to a weaker gas-holding capacity and less aerated structure (Dufour et al., 2024). The adhesiveness, which represents the force required to separate the sample from a surface, also showed an increasing trend with higher MP levels. This suggests that the incorporation of MP may contribute to a stickier and more adhesive texture, potentially affecting the mouthfeel and sensory perception of the product. Cohesiveness, a measure of the internal binding forces within the sample, exhibited a slight decrease at moderate MP levels (4%), but no significant differences were observed at higher levels compared to the control. This indicates that the addition of MP did not substantially impact the internal cohesion of the crumb structure. The elasticity of CSB remained unaffected by MP addition, suggesting that the ability of the product to regain its original shape after compression was not significantly influenced by the presence of microalgae powder.

The SV of CSB, an indicator of the overall expansion and lightness of the product, decreased significantly with MP levels above 4%. This reduction in SV can be attributed to the dilution of the gluten network by MP, which weakened the dough's gas-holding capacity and mechanical strength during proofing and steaming. Similar results were also observed in the CSB with the addition of quinoa flour or high-amylose corn starch (Wang et al., 2015; Wang et al., 2017). Additionally, the presence of dietary fibers in MP might have competed for water binding, leading to partial dehydration of the gluten network and consequent collapse of the structure, further contributing to the reduced specific volume (Zhou et al., 2021).

The observed changes in textural properties and SV highlight the importance of optimizing MP addition levels to achieve desirable sensory attributes while leveraging the nutritional benefits of microalgae incorporation. Excessively high MP levels may lead to an overly dense, hard, and chewy texture, which might not be favored by consumers. Therefore, striking a balance between texture, SV, and nutritional enhancement is crucial for the successful development of MP-enriched CSB products.

### 3.9 Effects of MP on nutrient composition of CSB

The addition of MP to CSB has significant effects on its nutrient composition, as shown in Table 2. The data indicate changes in calorie, protein, fat, and carbohydrate content with varying levels of MP. The total caloric value of CSB showed an increasing trend with increasing levels of MP addition. This can be mainly attributed to the relatively high protein and lipid contents in the microalgae powder itself.

**Table 2:** Effects of MP addition on the nutrient composition of CSB.

MP Addition	Calorie (kCal/100 g)	Protein (g/100 g)	Fat (g/100 g)	Carbohydrate (g/100 g)
0%	229.67 ± 2.89^b^	7.10 ± 0.10^b^	2.63 ± 0.06^c^	44.33 ± 1.02^a^
2%	228.67 ± 2.89^b^	7.10 ± 0.26^b^	3.23 ± 0.15^b^	43.07 ± 0.90^a^
4%	231.33 ± 2.31^ab^	7.47 ± 0.06^ab^	3.40 ± 0.26^b^	42.73 ± 0.38^a^
6%	236.33 ± 2.52^a^	7.57 ± 0.15^ab^	3.90 ± 0.17^a^	43.40 ± 1.51^a^
8%	236.67 ± 1.15^a^	7.80 ± 0.20^a^	3.93 ± 0.15^a^	42.47 ± 0.38^a^

***Note*:** Values are expressed as mean ± standard deviation. Different letters within the same column indicate significant differences (p < 0.05).

Protein is an essential nutrient for the formation of human tissues and organs. The results showed that MP additions of 8% significantly increased the protein content of CSB by 9.86% compared to the control group. This can be attributed to the presence of various nutritious proteins in *E gracilis*, such as amino acids, glycoproteins, and ribonucleoproteins. Microalgal proteins are often considered ideal plant-based protein supplements that can improve the nutritional value of bread and other baked goods (Fradique et al., 2010). As an important nutrient, lipids not only provide energy but also participate in various metabolic processes in the human body. The present study found that the addition of MP significantly increased the lipid content of CSB. This is due to the abundance of polyunsaturated fatty acids, such as linolenic acid, oleic acid, and palmitic acid, in *E. gracilis* cells (Teerawanichpan & Qiu, 2010). These fatty acids can not only increase the energy density of CSB but may also exert beneficial physiological functions, such as improving blood lipid levels and exhibiting anti-inflammatory effects (Lupette & Benning, 2020). Carbohydrates are the primary nutrient component in CSB. The results indicated that different levels of MP addition had little influence on the total available carbohydrate content of CSB. This may be due to the relatively low carbohydrate content in the microalgae powder itself, combined with the limited addition levels. However, *E. gracilis* is rich in various non-starch polysaccharides, such as paramylon, glucan, and algaenans, which are soluble dietary fibers that may have an impact on the physicochemical properties and shelf life of CSB (Barsanti et al., 2022).

Overall, the appropriate addition of MP can not only improve the physicochemical properties and flavor of CSB but also enhance its nutritional value, particularly by increasing the protein and lipid contents. Combining the nutrient-rich *E. gracilis* with traditional bread products could be a viable approach for developing functional foods. However, it is also necessary to balance the addition levels to avoid excessive amounts that may compromise product quality.

### 3.10 Correlation analysis

**Fig. 4:**
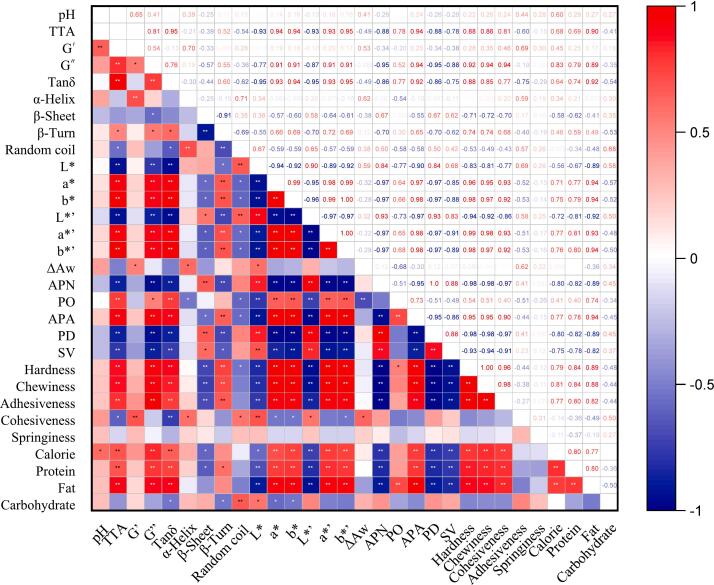
Correlation analysis of dough and CSB related indicators with different addition of MP. L*, a*, and b* are the colors of CSB core, and L*’, a*’, and b*’ are the colors of CSB crust. ∆Aw is the variation value of water activity over a period of seven days. The negative and positive correlations are represented by blue and red hues, respectively. The correlation coefficients cover the spectrum from −1 to 1. * and ** indicate that the correlation coefficient is significant at the *p*-value levels of 0.05 and 0.01, respectively. (For interpretation of the references to color in this figure legend, the reader is referred to the web version of this article.)

The correlation analysis between various properties of dough and CSB with different levels of MP addition provides valuable insights into how MP affects the overall characteristics of the final product. The correlation heat map (Fig. 4) illustrates both positive and negative correlations among different parameters, with the strength of these correlations represented by the intensity of the color gradient.

The rheological parameter G" was positively correlated with G' (*p* < 0.05) and tan δ (*p* < 0.01) in the dough, and this implies that changes in the viscoelastic behavior of the dough were interrelated. G' showed a significant positive correlation (*p* < 0.01) with the α-helix content in the secondary structure of dough proteins. Additionally, G" had a significant negative correlation (p < 0.05) with β-sheets and a significant positive correlation (p < 0.05) with β-turns. The correlations between rheological parameters and protein secondary structure indicate that the conformational changes in gluten proteins influenced the dough's viscoelastic properties.

The hardness, chewiness, and adhesiveness of CSB texture exhibited significant positive correlations (*p* < 0.01) with each other, but no significant correlations with cohesiveness or springiness were observed. Furthermore, hardness, chewiness, and adhesiveness showed significant positive correlations with a* (p < 0.01), b* (p < 0.01), a*’ (p < 0.01), b*’ (p < 0.01), and APA (p < 0.01), and significant negative correlations with L* (p < 0.01), L*’ (p < 0.01), APN (p < 0.01), PD (p < 0.01), and SV (p < 0.01). The strong positive correlations among hardness, chewiness, and adhesiveness of CSB texture suggest that these textural attributes were closely linked and influenced by similar factors. The negative correlations between these textural parameters and L*, SV, and pore characteristics (number and density) imply that a denser crumb structure with fewer and smaller pores resulted in harder, chewier, and more adhesive CSB. Conversely, the positive correlations with a*, b*, and APA suggest that larger pores and a more yellow/brown color were associated with increased hardness, chewiness, and adhesiveness.

These correlation analyses provide valuable insights into the interplay between the microalgae powder addition, dough rheology, protein structure, and the final quality attributes of the CSB, highlighting the complex interactions and their impact on the overall product characteristics.

## 4 Conclusions

The incorporation of *E. gracilis* powder to CSB impacted the dough properties, rheological behavior, and final product quality. At 2% MP, the gluten network was reinforced with improved protein structure. Higher MP levels (4–8%) adversely affected gluten and rheological properties. MP decreased CSB specific volume, pore number, but increased pore size, hardness, and chewiness. It imparted a yellow color and slower moisture loss during storage. MP effectively increased CSB's protein and lipid content, enhancing nutritional value. Optimizing MP level is crucial to achieve nutritional enhancement with desirable texture and sensory attributes. The addition of 2% MP can balance nutrition and the quality of the final product. Future research should focus on pretreatment of MP before its addition and the effects of other different edible MP, combined with other ingredients, to improve structural stability and achieve the optimum texture, flavor, and nutrition of MP-enriched CSB.

## Funding

This research is supported by the 10.13039/501100001809National Natural Science Foundation of China (Grant No. 22208285), the High-Level Talent Introduction Fund of Yangzhou University (Grant No. 137012779), the Innovative Talent Program of “Golden Phoenix of the Green City” (Grant No. YZLYJF2022YXBS169), and the Research Fund of Sichuan Cuisine Development Research Center, Philosophy and Social Sciences Key Research Base of Sichuan Province (Grant No. CC23Z06).

## CRediT authorship contribution statement

**Jiangyu Zhu:** Writing – review & editing, Writing – original draft, Visualization, Validation, Supervision, Software, Methodology, Investigation, Funding acquisition. **Yifei Cai:** Writing – original draft, Visualization, Validation, Software, Methodology, Investigation, Formal analysis. **Yan Xu:** Visualization, Project administration, Formal analysis, Data curation. **Xiao Wei:** Resources, Formal analysis, Data curation. **Zhengfei Yang:** Supervision, Methodology. **Yongqi Yin:** Visualization, Supervision, Resources. **Minato Wakisaka:** Writing – review & editing, Writing – original draft, Supervision, Project administration, Funding acquisition. **Weiming Fang:** Supervision, Project administration.

## Declaration of competing interest

The authors declare that they have no known competing financial interests or personal relationships that could have appeared to influence the work reported in this paper.

## Data Availability

Data will be made available on request.

## Appendix A Supplementary data

Supplementary material Image 1
