# Supplementary material for: Effects of heterotrophic Euglena gracilis powder on dough microstructure, rheological properties, texture, and nutritional composition of steamed bread
Source: Food Chem X. 2024 Aug 20;23:101754. doi: 10.1016/j.fochx.2024.101754 (PMC11388338; doi:10.1016/j.fochx.2024.101754)
Supplement: Supplementary file 1 — Supplementary material [file mmc1.docx]

**Effects of Heterotrophic *Euglena gracilis* Powder on Dough Microstructure, Rheological Properties, Texture, and Nutritional Composition of Steamed Bread**

Jiangyu Zhu^1,*^, Yifei Cai^1^, Yan Xu^1^, Xiao Wei^1^, Zhengfei Yang^1^, Yongqi Yin^1^, Minato Wakisaka^2,*^, Weiming Fang^1,*^.

^1^ School of Food Science and Engineering, Yangzhou University, No. 196 Huayang West Road, Hanjiang District, Yangzhou 225127, China;

^2^ Food Study Centre, Fukuoka Women’s University, 1-1-1 Kasumigaoka, Fukuoka 813-8529, Japan.

*Correspondence: 008051@yzu.edu.cn (J. Z.).

**Table S1** Effects of different addition amounts of MP on the pH and TTA of the dough.

| MP addition | pH | TTA (mL) |
| --- | --- | --- |
| 0% | 5.30±0.02^a^ | 1.37±0.12^d^ |
| 2% | 5.26±0.04^a^ | 2.53±0.15^c^ |
| 4% | 5.25±0.03^a^ | 2.83±0.12^bc^ |
| 6% | 5.32±0.04^a^ | 3.30±0.26^ab^ |
| 8% | 5.29±0.02^a^ | 3.50±0.30^a^ |

**Note:** Different lowercase superscripts in the same column represent significant differences.

**Table S2** Power law parameters derived from frequency sweep tests for dough samples with varying algae content.

| MP addition | Storage Modulus (G') | | Loss Modulus (G") | |
| --- | --- | --- | --- | --- |
|  | n' | K' (kPa·s^n'^) | n'' | K''(kPa·s^n''^) |
| 0% | 0.333±0.003^e^ | 12.31±0.35^a^ | 0.217±0.002^d^ | 6.80±0.22^b^ |
| 2% | 0.394±0.002^d^ | 9.62±0.47^b^ | 0.269±0.003^c^ | 6.53±0.57^b^ |
| 4% | 0.418±0.001^c^ | 8.32±0.50^c^ | 0.293±0.004^b^ | 6.31±0.35^b^ |
| 6% | 0.429±0.003^b^ | 9.77±0.32^b^ | 0.298±0.002^ab^ | 7.66±0.43^a^ |
| 8% | 0.443±0.004^a^ | 10.19±0.15^b^ | 0.304±0.004^a^ | 8.28±0.38^a^ |

**Note:** Values are expressed as mean ± standard deviation. Different letters within the same column indicate significant differences (p<0.05).

**Table S3** Effects of different inclusion levels of MP on the color of CSB.

| MP addition | Core of CSB | | | | Crust of CSB | | | |  |
| --- | --- | --- | --- | --- | --- | --- | --- | --- | --- |
|  | L* | a* | b* | △E | L* | a* | b* | △E |  |
| 0% | 82.49±0.58^a^ | 1.49±0.17^a^ | 14.06±0.50^a^ | - | 81.59±0.83^a^ | 1.31±0.02^a^ | 12.46±0.05^a^ | - | |
| 2% | 78.14±0.03^b^ | 3.27±0.05^b^ | 20.05±0.29^b^ | 5.94 | 79.19±0.21^b^ | 2.29±0.08^b^ | 17.8±0.28^b^ | 7.61 | |
| 4% | 77.39±0.66^b^ | 4.07±0.07^c^ | 25.01±0.35^c^ | 11.15 | 77.04±0.73^c^ | 3.58±0.04^c^ | 22.38±0.09^c^ | 12.35 | |
| 6% | 76.8±0.24^bc^ | 4.86±0.28^d^ | 28.8±0.62^d^ | 15.21 | 75.5±0.54^cd^ | 4.47±0.04^d^ | 26.03±0.07^d^ | 16.16 | |
| 8% | 75.84±0.79^c^ | 6.13±0.12^e^ | 32.53±0.10^e^ | 18.30 | 74.99±0.59^d^ | 5.17±0.05^e^ | 29.09±0.19^e^ | 20.17 | |

**Note:** Values are expressed as mean ± standard deviation. Different letters within the same column indicate significant differences (p<0.05).

**Table S4** Effects of different inclusion levels of MP on pore distribution in CSB cross-section.

| MP addition | APN | PO (%) | APA (10^-3^·cm^2^) | PD (pcs·cm^-2^) |
| --- | --- | --- | --- | --- |
| 0% | 265.00±2.00^a^ | 22.67±0.47^c^ | 7.70±0.17^d^ | 29.44±0.22^a^ |
| 2% | 250.67±7.09^b^ | 26.31±0.73^b^ | 9.44±0.01^c^ | 27.85±0.79^b^ |
| 4% | 228.33±5.51^c^ | 25.71±0.11^b^ | 10.14±0.23^b^ | 25.37±0.61^c^ |
| 6% | 213.67±1.53^d^ | 28.43±0.48^a^ | 11.97±0.27^a^ | 23.74±0.17^d^ |
| 8% | 185.67±5.86^e^ | 25.72±0.32^b^ | 12.47±0.27^a^ | 20.63±0.65^e^ |

**Note:** Values are expressed as mean ± standard deviation. Different letters within the same column indicate significant differences (p<0.05).

**Fig. S1** Schematic diagram of pore distribution in CSB Cross-section. (a-e: 0%, 2%, 4%, 6% and 8% MP additions, respectively)

**Fig. S2** Water activity changes of CSB with different levels of MP during storage.
